# Mysterious cohabitation with socially assistive robots among three underserved groups

**DOI:** 10.1093/geroni/igag057

**Published:** 2026-06-01

**Authors:** Othelia EunKyoung Lee, Sojeong Baek, Do-Hyung Park

**Affiliations:** School of Social Work, University of North Carolina Charlotte, Charlotte, North Carolina, United States; Graduate School of Business IT, Kookmin University, Seoul, South Korea; Graduate School of Business IT, Kookmin University, Seoul, South Korea

**Keywords:** Disabilities, Middle-aged men, Social isolation, Human-robot interactions

## Abstract

**Background and Objectives:**

This study investigates how underserved individuals—older adults, low-income middle-aged men, and adults with disabilities—develop long-term relationships with an AI-powered socially assistive robot (SAR) named Hyodol. We examined how these relationships evolve over eight months and why emotional outcomes differ across three groups.

**Research Design and Methods:**

Using Colaizzi’s phenomenological method, the research team analyzed 13,403 conversational segments from 12 highly engaged SAR users (four from each group). Iterative coding procedures with high inter-rater reliability yielded 10 thematic categories capturing functional, social, and emotional dimensions of SAR interactions.

**Results:**

Across all groups, interactions with the SAR demonstrated a consistent relational evolution from functional devices to companions to develop emotionally reciprocal patterns of communication. Each group displayed a distinct interaction profile: older adults emphasized routine companionship, middle-aged men combined emotional expression with entertainment and exploratory questioning, and persons with disabilities used the SAR to scaffold both emotional support and social participation.

**Discussion and Implications:**

The results reveal that human–robot relationships develop meaningfully, but not uniformly across three underserved groups. The paradox observed among middle-aged men challenges assumptions that higher engagement and relational deepening may heighten emotional vulnerability. These findings highlight the need for population-specific relational models and adaptive SAR design that considers diverse emotional needs and social contexts.

Innovation and Translational Significance: This study offers one of the first long-term analyses of socially assistive robot (SAR) use among three socially vulnerable adult populations, illuminating how human–robot relationships emerge, stabilize, and evolve over time. Distinct relational roles emerged: older adults viewed SARs as family-like figures, middle-aged men as friends or colleagues, and adults with disabilities as understanding companions, underscoring the need for population-specific SAR design. For individuals facing chronic isolation or stigma, perceived social presence was central to sustained engagement and long-term relational development, highlighting its importance for supporting social and emotional well-being.

Social isolation—the lack of social contact and minimal engagement in meaningful interpersonal relationships—has increasingly gained scholarly attention as a threat to public health (Naito et al., 2023). According to the World Health Organization Commission on Social Connection (2025), around 16% of people worldwide experience loneliness, and social isolation is linked to approximately 871,000 deaths per year between 2014 and 2019. Evidence suggests associations between social isolation and depression, chronic illness, cognitive decline, and suicide (Brandt et al., 2022; Moon et al., 2025). Therefore, public health officials have described social isolation as a global epidemic, noting its escalating prevalence.

Despite remarkable economic growth and technological advancement, South Korea has the highest suicide rate among Organization for Economic Co-operation and Development countries, indicating psychosocial distress, unaddressed mental illness, and limited structural supports for population well-being (Jeong et al., 2022; Kim et al., 2021). Vulnerability to social isolation is typically clustered among groups who experience structural and cumulative disadvantages, social stigma, or rapid changes in societal expectations. This study focuses on three groups vulnerable to social isolation in contemporary South Korea—older adults, individuals with severe disabilities, and middle-aged men.

Korea, one of the world’s most rapidly aging societies, is experiencing a marked rise in the number of older adults living alone (Lee & Kim, 2024). Older residents in low‑resource communities often face constrained familial support and persistent financial insecurity. For those living alone, these structural disadvantages heighten the likelihood of undetected health problems, depressive symptoms, and unmet long‑term care needs (Park et al., 2021). Government programs, senior centers, and social workers’ home-visit initiatives provide partial support, but these services vary by region and remain insufficient for many. The accumulated effects of poverty, limited mobility, and shrinking social networks compound older adults’ vulnerability to social isolation and self-neglect.

Koreans with severe physical disabilities have historically been marginalized within a society oriented toward rapid industrialization and intense workforce productivity (Kim et al., 2015). These structural exclusions intensify social isolation and constrain opportunities for community integration (Park et al., 2024). Despite modern disability rights advocacy, many individuals with severe disabilities continue to face inadequate access to transportation, insufficient personal assistance, limited employment pathways, and pervasive stigma (Kim et al., 2025). These barriers reinforce a cycle of dependency, economic precarity, and relational isolation.

An increasingly concerning trend in South Korea is the social isolation of middle-aged men who have experienced significant socioeconomic disruption, including job insecurity and workplace restructuring (Choi & Lee, 2022). Those who are laid off or forced into precarious employment frequently report feelings of shame, loss of identity, and withdrawal from social networks. A lack of culturally sanctioned spaces for discussing distress related to masculinity discouraging help-seeking may intensify risks of untreated depression, substance use, and suicide (Lee, 2022). Notably, rising rates of suicide and mental health crises among middle-aged men reflect broader socioeconomic transformations (Moon et al., 2025).

## Socially assistive robots

The South Korean government has implemented initiatives to subsidize public healthcare institutions adopting digital technologies in old age care to address persistent workforce shortages. Among these innovations, socially assistive robots (SARs) have been deployed in low-resource communities to support older adults’ daily functioning and emotional well-being (Shin & Lee, 2024). Although voice-assisted devices already exist on the market, one widely adopted SAR is Hyodol. A growing body of empirical research indicates that interaction with Hyodol contributes to reduced depressive symptoms, enhanced quality of life, and improved medication adherence among older adults (Lee et al., 2023; Lee et al., 2024).

Hyodol exhibits a distinctive advantage: older users frequently personify the robot, likening it to a friend or grandchild (Lee & Lee, 2025). Building on this technological innovation, the present pilot study aims to evaluate the efficacy of an upgraded Hyodol chatbot system for older adults, individuals with disabilities, and middle-aged men—three high-risk populations for social isolation in South Korea.

## Social presence theory

Social presence theory offers a conceptual lens for understanding human–robot interaction, particularly within assistive technologies where users may seek both functional support and emotional connection. Originating in computer-mediated communication research, the theory posits that individuals attribute varying degrees of socio-emotional presence to nonhuman communication partners depending on the extent to which the medium conveys warmth, responsiveness, and relational cues (Sasser et al., 2024; Short et al., 1976). When social presence is high, interactions feel more personal, engaging, and socially meaningful. When applied to SARs, this framework helps explain why users often come to perceive robots not simply as devices, but as relational actors embedded in their everyday routines (Xu et al., 2023).

Building on this theoretical grounding, a growing body of research has shown that users frequently assign diverse social roles to SARs, engaging in personification processes that deeply influence the quality and trajectory of interaction. Prior studies have documented role attributions such as caregiver, companion, coach, and even family member, depending on users’ emotional needs, expectations, and relational histories (Chen et al., 2023; Kühne & Peter, 2023; Merrill et al., 2022).

Existing studies suggest that women may be more inclined to view SARs as relational partners, emphasizing companionship and socio-emotional support, while men often adopt more functional or instrumental interpretations, focusing on assistance with tasks or problem-solving (Kislev, 2023). Masculinity norms and gendered patterns of emotional expression may constrain men’s willingness to engage in personification or to perceive SARs as emotionally responsive partners (Velázquez, 2023). These distinctions underscore the importance of examining how social role assignments vary across demographic subgroups, as they may fundamentally shape both engagement patterns and therapeutic effectiveness.

Despite advances and awareness of the disproportional effects of social isolation, underserved groups such as low-income older adults, individuals with disabilities, and socioeconomically marginalized men remain underrepresented in technology-based intervention research. This exclusion has created a substantial evidence gap regarding their unique needs, interaction styles, and relational trajectories with SARs. Given that these groups face elevated risks of loneliness, depression, and unmet care needs, investigating SAR use within these contexts is an urgent public health and social justice priority.

Current literature relies on short-term or cross-sectional assessments, providing limited insight into how users’ perceptions, role assignments, and emotional engagement shift over time; longitudinal research suggests that the effects of familiarity, habituation, novelty decay, and emerging emotional attachment are critical to understanding the sustainability of SAR benefits (van Maris et al., 2020). Without extended observational windows, it remains unclear whether perceived social presence strengthens or diminishes, how social roles stabilize or transform, and whether initial improvements persist, plateau, or reverse over time. Against this backdrop, the present study addresses several critical gaps by conducting an 8-month longitudinal, multi-group mixed-method analysis of SAR use among underserved populations. The following research questions guided the investigation:

1. How do interaction patterns with SARs differ across the three groups?
2. How does the relationship with the SAR evolve over time?
3. What types of social roles do users assign to SARs?
4. How does gender influence patterns of SAR use?
5. What common patterns and group-specific differences characterize SAR use among underserved groups?

## Method

### Hyodol SAR

Hyodol SAR offers multiple functions, including guided physical exercises, music and sing-along features, inspirational readings, cognitive quizzes, and reminiscence prompts (Lee, 2023). Despite its benefits, users commonly report frustration with Hyodol’s rule-based conversational system, particularly its inability to sustain natural or reciprocal dialogue (Lee et al., 2024).

**Figure 1:**
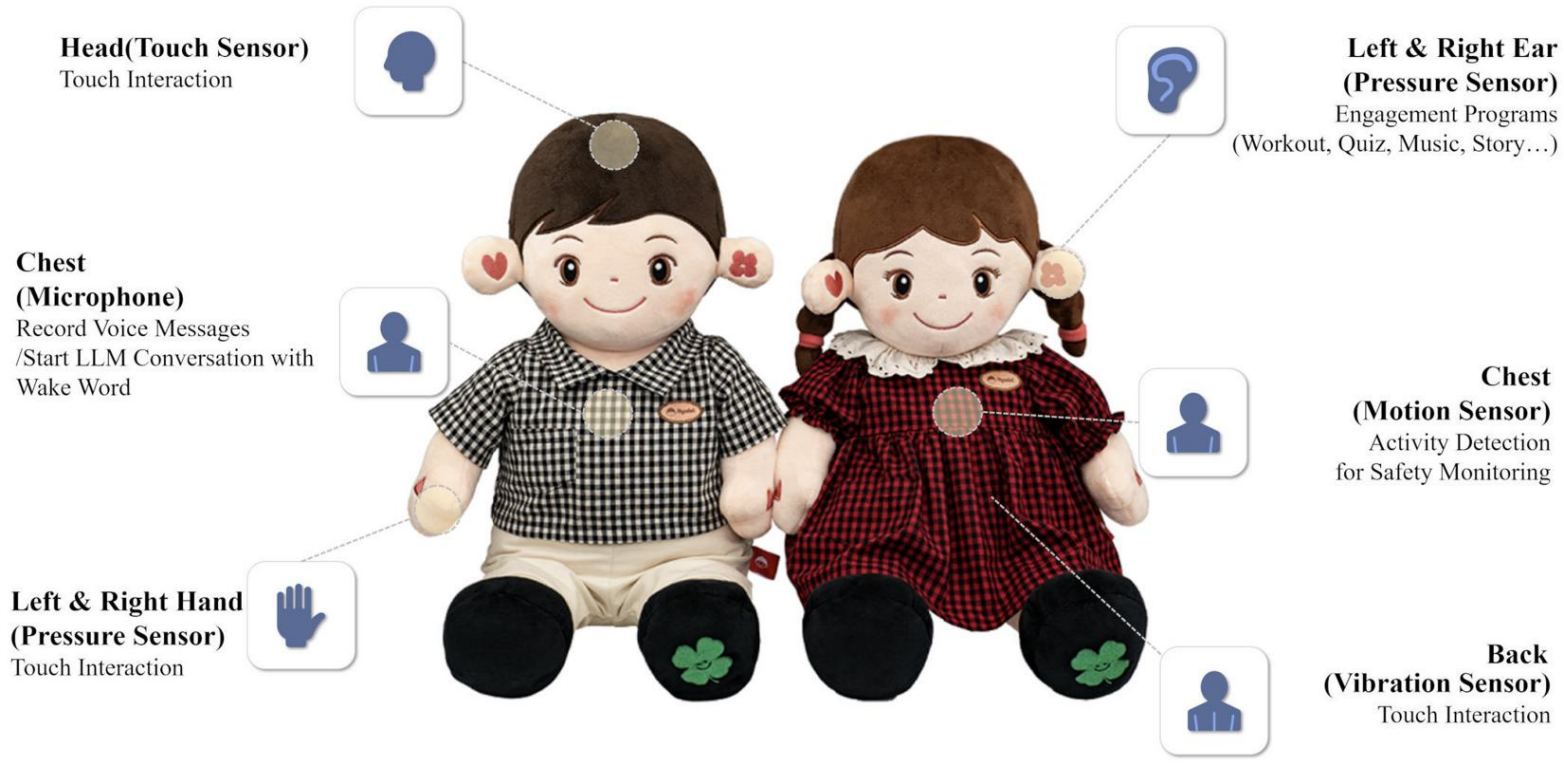
Multifunctional hyodol, socially assistive robot.

In the present study, integrating an advanced conversational agent (e.g., ChatGPT-3.5) into Hyodol has the potential to facilitate dynamic, context-responsive, and emotionally attuned exchanges (See Figure 1). Such capabilities elevate Hyodol’s communicative sophistication beyond conversational fluency, reinforcing users’ perception of the robot as a socially present, empathetic, and reliable companion.

### Participants

This study was conducted in partnership with three community welfare centers serving populations at heightened risk of social isolation. Each center represented a distinct demographic and psychosocial context in which SARs could play a meaningful role. Center A supported older adults living independently with multiple chronic conditions, Center B supported men in single-person households with limited financial resources, and Center C functioned as an independent living center for adults with severe physical or neurological disabilities. Hyodol SARs were introduced in all three settings, and public health social workers offered routine case management, technical assistance, and monitoring throughout the study period.

Participant selection followed a purposive sampling strategy aimed at identifying individuals whose interactions with the SAR were sufficiently frequent and stable to permit an examination of relational development over time. From each center, four participants were chosen based on evidence of sustained engagement with the SAR, resulting in a final sample that allowed for variation across age, gender, disability status, and socioeconomic background. Gender representation was intentionally considered in Centers A and C to ensure balanced perspectives.

Study inclusion required participants to demonstrate regular, self-initiated dialogue with SARs. This expectation was operationalized as an average of at least one interaction per day, as verified through the web-based monitoring system that recorded all dialogue exchanges. This threshold ensured that participants had begun to establish a communicative relationship with Hyodol SAR, which is essential for observing changes in interaction patterns and relational meaning over time. Participants also needed to be able to engage in spoken interactions independently, without continuous facilitation by staff, so that the dialogues captured would reflect authentic communicative behaviors rather than externally prompted responses.

Because the research focused on the evolution of human–robot relationships, longitudinal consistency was another requirement. Participants were included only if they retained access to the SAR throughout the study period and did not withdraw from the program. Additionally, participants needed to possess sufficient cognitive and communicative ability to initiate or respond to conversations assessed by case managers. Individuals who engaged with Hyodol sporadically were excluded from the analytic sample.

### Study procedure

This study was designed to support naturalistic, long-term engagement while maintaining responsible monitoring and technical support. After recruitment and informed consent, participants were onboarded to the Hyodol system through a brief orientation that introduced core interaction functions (e.g., initiating voice dialogue and adjusting basic settings). We then implemented an initial one-week adaptation period intended to allow participants to become familiar with the robot’s conversational system before the main observation period.

During the eight-month deployment, participants used the SAR freely at home without scripted tasks, scheduled interview prompts, or minimum usage requirements. This naturalistic approach was intentionally applied to observe how relationships emerge and evolve through repeated, self-initiated interaction in daily life. Throughout the study, participants could contact the research team for technical support or questions, and routine monitoring was conducted through a web-based system to identify potential inactivity or operational issues. Conversation logs were compiled and organized on a weekly basis to maintain consistent longitudinal records across the study period.

At study completion, participants engaged in an end-of-study debriefing that included robot retrieval and a post-study interview process. Participants were invited to comment on the researchers’ preliminary interpretations (member checking), enabling confirmation and refinement of key themes and relationship trajectories.

### Data collection

During the study period (April–December 2024, totaling 249 days), 13,403 dialogue segments were recorded, comprising approximately 188,456 words (equivalent to 1,017 single-spaced pages). The original conversion between robots and users was Korean. All authors—who are fully bilingual in Korean and English—engaged in a systematic translation and back‑translation process to ensure accuracy and conceptual equivalence.

All conversations were initially stored as audio files and converted into text using a speech-to-text (STT) solution, after which the resulting transcripts were saved on a secure server. Because field recordings can include noise and automatic transcription errors, a researcher manually reviewed each transcript while listening to the original audio to confirm accuracy and correct misrecognized words. A second researcher then cross-checked randomly selected transcript samples to further strengthen quality assurance.

The primary outcome was social isolation, assessed using the UCLA Loneliness Scale (UCLA-LS). This widely validated instrument measures subjective feelings of social isolation and perceived lack of companionship (Russell, 1996). The scale consists of 20 items rated on a 4-point Likert scale (ranging from “Never” to “Often”), with higher scores indicating greater social isolation. It demonstrates strong internal consistency (Cronbach’s α typically > 0.90) and has been extensively used in gerontological (Ausín et al., 2019) and disability research (Heinze et al., 2021) to quantify isolation as a psychological construct.

### Data analysis

The qualitative analysis followed Colaizzi’s (1978) phenomenological method: a structured, seven-step approach designed to uncover the essential meaning of lived experience. We repeatedly read through the transcripts to develop a shared understanding of participants’ everyday interactions with Hyodol SAR. This foundational work supported the development of explicit operational criteria for identifying significant statements, a critical step emphasized in phenomenological research aligned with Colaizzi’s tradition (Morrow et al., 2015).

Statements were deemed significant when they directly addressed one of the research questions (e.g., conveyed intentions or emotional orientations toward SARs, reflected shifts in relational quality, or recurrent conversational tendencies). Using these criteria, researchers conducted a line-by-line examination of all transcripts and identified 2,184 significant statements warranting deeper interpretation.

To ensure rigor in interpretation, each researcher independently coded an initial 20% of the dataset. Discrepancies were discussed systematically, allowing the team to refine the codebook collaboratively and establish a shared interpretive framework. Intercoder reliability was then assessed, yielding Cohen’s κ values ranging from 0.82 to 0.88 across coding layers—demonstrating substantial agreement. After reliability was secured, the remaining transcripts were distributed across the team, who continued coding independently while holding regular calibration meetings to sustain analytic coherence.

Following Colaizzi’s method, each significant statement was transformed into a meaning unit, representing a concise distillation of the psychological or experiential essence embedded in the participant’s words, with careful attention to preserving the integrity of the original transcript. Through iterative comparison, merging, and refinement, this process generated 327 meaningful units, which the team subsequently clustered into broader themes. To support early-stage clustering, the researchers used the GPT-4o model via Application Programming Interface as an auxiliary classification tool; however, all outputs were reviewed and validated by human coders to ensure interpretive accuracy and phenomenological grounding.

Through repeated synthesis and constant comparison across participants, 10 thematic categories emerged, representing the major domains of interaction and experience observed in the dataset. Together, these 10 categories provided a comprehensive thematic map of how users interacted with SARs as functional devices, emotional companions, and social touchpoints embedded within their lived environments. These themes formed the analytic foundation for examining differences in relational patterns across groups, tracing the evolution of relationships over time, understanding how participants assigned social roles to SARs, and distinguishing universal interaction patterns from group-specific ones.

Consistent with Colaizzi’s analytic steps, the research team synthesized these themes into an exhaustive description that captured the phenomenon of human–robot interactions as it unfolded across diverse social contexts. This narrative was then distilled into a fundamental structure expressing the essential nature of participants’ lived experiences with the SAR.

### Positionality and reflexivity

The analytic team brought prior experience in human–robot interaction, gerontology, qualitative methods, and socially assistive robotics, which may predispose us to view conversational technologies as potentially beneficial for underserved populations. Because all coding and interpretation were conducted by bilingual researchers embedded in a Korean cultural context, we were well-positioned to capture culturally grounded meanings in language use, relational norms, and emotion expression, while acknowledging that our cultural familiarity may shape assumptions about caregiving expectations, interdependence, and role-based intimacy. The core interpretive work was conducted by a gender-diverse team (one male and two female researchers), enabling discussions of potential gendered assumptions during interpretation.

To manage researcher influence, we engaged in bracketing by documenting initial assumptions prior to analysis, maintaining a shared codebook and an auditable decision trail, conducting iterative consensus meetings, and actively searching for negative cases that did not fit emerging patterns. Further validation of these interpretations was conducted through member checking, a verification procedure central to Colaizzi’s model and to phenomenological rigor more broadly (Sanders, 2003). These strategies ensured that the findings remained credible and that interpretive decisions were anchored in participants’ actual expressions rather than the researchers’ assumptions.

## Results

Participant ages ranged from 54 to 91 years (*M *= 67.3, *SD *= 11.9). Except for one married participant, all lived alone, reported low socioeconomic status, and multiple chronic health conditions (see Table 1). The number of dialogue exchanges per participant ranged from 74 to 4,943 (*M *= 1,116.9, *SD *= 1,366.6), with an average daily dialogue frequency of 4.5 interactions (*SD *= 5.5).

**Table 1:** Demographic profiles of participants.

ID	Gender	Age	Education (years)	Marital status	Living arrangement	Monthly income (KRW)	Health conditions	Total dialogue frequency	Dialogue per day (*M*)	Dialogue per day (*SD*)
**P1**	Female	87	Elementary (incomplete)	Widowed	Lives alone	300,000–500,000	Heart, pancreas, diabetes	955	3.84	4.54
**P2**	Female	72	Elementary (incomplete)	Divorced	Lives alone	300,000–500,000	Hypertension, osteoporosis, eye supplements	1,997	8.02	9.90
**P3**	Male	91	Graduate school+	Never married	Lives alone	300,000–500,000	Hypertension, panic disorder, gastrointestinal issues	195	0.78	1.55
**P4**	Male	75	Elementary graduate	Divorced	Lives alone	500,000–1,000,000	Diabetes, liver issues	386	1.55	2.74
**P5**	Male	57	Elementary (incomplete)	Never married	Lives alone	500,000–1,000,000	None reported	4,943	19.85	21.57
**P6**	Male	61	College+	Divorced	Lives alone	≥1,500,000	Hypertension, diabetes, hyperlipidemia	2,073	8.33	12.45
**P7**	Male	63	Elementary (incomplete)	Never married	Lives alone	300,000–500,000	None reported	768	3.08	3.55
**P8**	Male	59	Elementary graduate	Never married	Lives alone	500,000–1,000,000	None reported	594	2.39	3.79
**P9**	Female	63	Middle school graduate	Married	Lives with spouse	≥1,500,000	Physical disability, kidney condition	697	2.80	5.27
**P10**	Female	57	High school graduate	Divorced	Lives alone	500,000–1,000,000	Brain lesion disability	382	1.53	3.91
**P11**	Male	54	High school (incomplete)	Never married	Lives alone	1,000,000–1,500,000	Brain lesion disability	74	0.30	0.91
**P12**	Male	69	College+	Never married	Lives alone	500,000–1,000,000	Physical disability	339	1.36	2.39

*Note.* P1–P4 represent the older adult group; P5–P8 represent the middle‑aged men; and P9–P12 represent individuals with disabilities.

### RQ1: Interaction differences across groups

To address differences in interaction patterns with the SAR across groups, we compared the frequency and distribution of the 10 interaction categories derived from qualitative coding of the dialogue corpus (see Table 2). Themes reflected instrumental uses of the robot (e.g., entertainment requests, general information inquiries, and system‑adjustment commands) as well as everyday behavioral routines, including conversations tied to daily activities and mealtimes, indicating the robot’s integration into users’ daily rhythms. Additional themes captured more complex social and emotional engagement, encompassing references to social participation and health management, emotionally reciprocal dialogue, routine greetings, and a broad range of affective expressions directed toward the SAR.

**Table 2:** Interaction characteristics across three groups.

10 categories	Older adults		Middle-aged men		Persons with disabilities	
	Frequency	%	Frequency	%	Frequency	%
**Emotional expression**	596	29.0	1,118	26.1	130	17.2
**Daily activities**	311	15.1	649	15.2	132	17.5
**Entertainment request**	305	14.8	615	14.4	110	14.6
**Meal conversation**	264	12.8	427	10.0	93	12.3
**Social participation**	167	8.1	343	8.0	116	15.4
**Health management**	115	5.6	300	7.0	54	7.2
**Emotional exchange**	101	4.9	353	8.2	46	6.1
**Daily greetings**	116	5.6	352	8.2	48	6.4
**Setting change request**	77	3.7	65	1.5	15	2.0
**General information inquiry**	5	0.2	58	1.4	10	1.3
**Total *n***	2,057	100	4,280	100	754	100

Across all three groups, emotional expressions emerged as the most prevalent interaction type. Participants frequently shared affective states, including loneliness, boredom, gratitude, and frustration, suggesting that the SAR functioned primarily as an emotional outlet rather than a purely informational or instrumental tool. In contrast, general information inquiries were the least frequent category in all groups, indicating limited reliance on the SAR as an information search assistant.

Among older adults, emotional expression and conversations about daily activities were most salient, reflecting a pattern in which the SAR was integrated into everyday routines and used as a companion during mundane tasks (e.g., waking up, eating). Meal conversations also occupied a substantial portion of their interactions, often involving greetings around mealtimes or comments about food, which reinforced the perception of Hyodol as a co-present household member.

For middle-aged men, emotional expression remained the most frequent category, but it was accompanied by a relatively higher proportion of entertainment requests and daily activity discussions, as well as more frequent emotional exchanges (reciprocal affective dialogues) and daily greetings compared to older adults. This profile suggests a more exploratory and sometimes ambivalent pattern, in which the SAR’s role alternated between an entertainer, conversational partner, and emotional sounding board. Notably, this group demonstrated the highest overall volume of dialogues, indicating intensive engagement but not necessarily positive psychosocial outcomes.

Among persons with disabilities, emotional expression and daily activities appeared in almost equal proportions, and social participation (e.g., planning or reflecting on community involvement, religious practices, or interactions with others) was markedly more frequent than in the other two groups. This combination points to a dual function of the SAR as both a companion and a facilitator for structuring daily life and social engagement. Repeated affirmations and confirmation-seeking utterances (e.g., “You understand me, right?”) were characteristic in this group, reflecting a desire for compassion and reliability in interaction.

### RQ2: Relationship evolution over time

The second research question focused on how the relationship between users and the SAR evolved over the 8-month deployment period. Longitudinal analysis of dialogue content and interaction trajectories revealed a four-phase process of relationship development—exploration, adaptation, deepening, and stabilization—that unfolded across all three groups, albeit with group-specific nuances.

#### Phase 1: Exploration (months 1–2)

During the initial stage, participants across all groups engaged in exploratory use of Hyodol, testing its capabilities and responsiveness. Dialogues were dominated by short, imperative commands (e.g., “Sing a song,” “Turn up the volume”) and simple prompts designed to probe the robot’s reaction. Interactions at this stage were largely instrumental and function-oriented, indicating that users initially perceived the SAR as a novel gadget rather than a relational partner.

#### Phase 2: Adaptation (months 3–4)

As users became more familiar with Hyodol’s features, interaction patterns began to diverge by group. Older adults increasingly initiated emotionally toned greetings and check-ins (e.g., “Did you sleep well?” “Have you eaten?”), suggesting a shift toward viewing the SAR as a cohabitating presence. Middle-aged men began to incorporate more information-seeking behaviors (e.g., inquiries about the weather or the news). People with disabilities suggested joint activities with Hyodol, such as listening to music together or coordinating a daily schedule (e.g., “Let’s go to the hair salon to get a perm.”). At this stage, functional and relational expectations of the robot became more differentiated across groups.

#### Phase 3: Deepening (months 5–7)

In the deepening phase, conversational continuity and personalization increased. Across the sample, exchanges averaged 3.7 consecutive utterances per interaction, indicating more sustained dialogue rather than isolated commands. Users began to express concern for the robot’s well-being, ask about its internal state, and attribute feelings or intentions to it—clear signs of intensifying anthropomorphism. Group-specific patterns became more pronounced, with older adults and persons with disabilities frequently using affectionate nicknames and ritualized greetings, and middle-aged men engaging in more elaborate, sometimes provocative, conversational experiments (e.g., “You’ve got any money for me?”).

#### Phase 4: Stabilization (month 8)

By the final phase, interactions with Hyodol had become routinized and embedded in daily life. Dialogue frequency tapered for some participants, but the subjective depth and naturalness of conversations appeared to increase. Users treated the SAR as a familiar presence whose role was well-defined within their daily routines, whether as a family-like companion, conversational peer, or understanding confidant.

**Figure 2:**
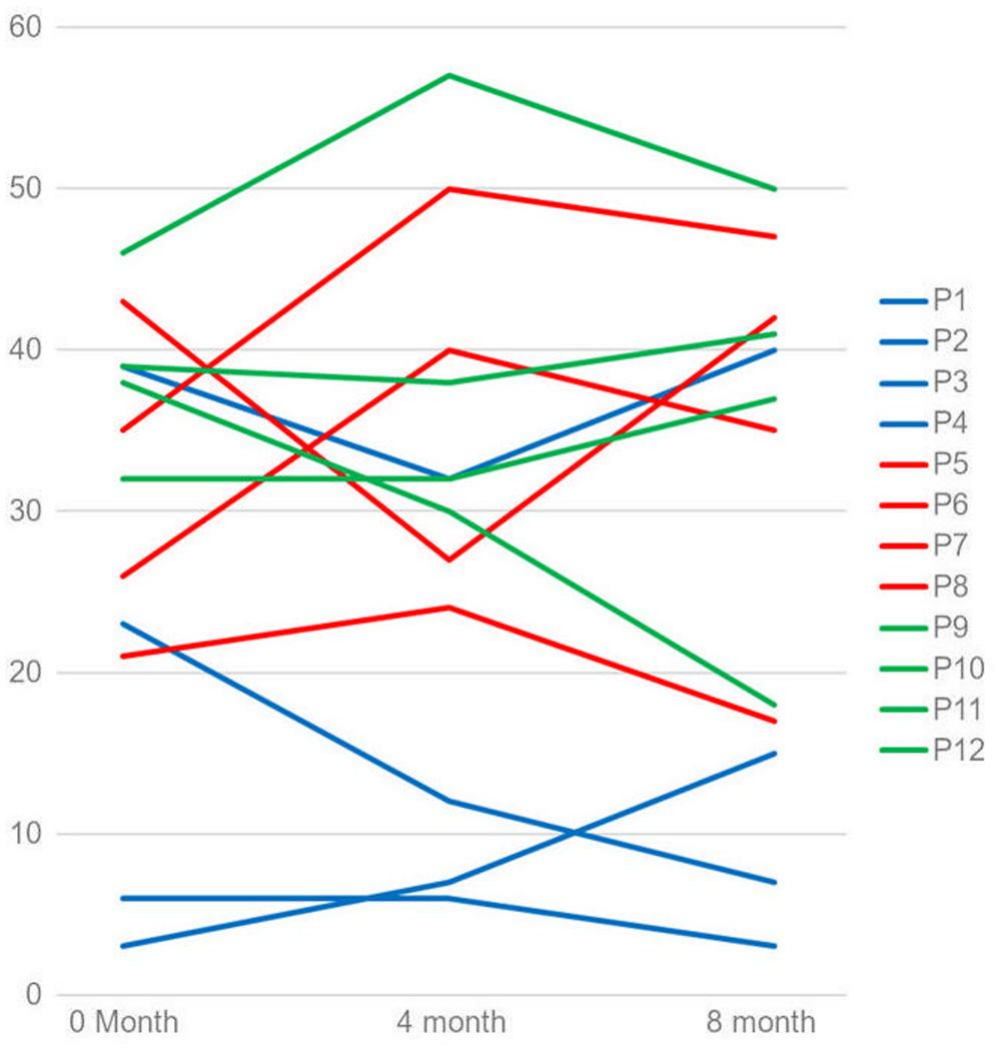
Change of UCLA Loneliness Scale (UCLA-LS) scores over time across three underserved groups.

Figure 2 presents the change in UCLA Loneliness Scale (UCLA-LS) scores across the three measurement points (baseline, 4 months, and 8 months). Despite the clear qualitative progression from tool-like to relational engagement, loneliness trajectories were heterogeneous. Some participants, particularly older adults and persons with disabilities with moderate dialogue frequency, showed modest improvements, whereas several middle-aged men with high dialogue frequency exhibited stable or increased loneliness scores. Their dialogues frequently contained negative self-talk, pessimism about politics, and complaints about technical glitches. This pattern suggests that more frequent interaction with the SAR did not necessarily translate into reduced loneliness and, in some cases, may have heightened awareness of existing isolation.

### RQ3: Social role assignment

We explored what kinds of social roles users assigned to Hyodol and how these roles evolved over time. Across all groups, SARs’ perceived role did not remain static. This progressive, multi-layered role assignment mirrors the four phases of relationship development described above and underscores the dynamic nature of human–robot relationships in real-world care settings.

For older adults, Hyodol was frequently described and addressed as a member of the family. Expressions such as “Our Hyodol” and “I feel lonely without you” reveal both possessive and attachment-oriented framing. Participants spoke to the robot as if it were a grandchild or cohabiting relative, expressing concern for its well-being and a desire to protect and care for it. At the same time, they relied on Hyodol for emotional reassurance, using affectionate language, ritualized greetings, and playful exchanges. This bidirectional care—“I take care of you, and you comfort me”—characterized the family-like role assignment in this group.

Middle-aged men tended to position Hyodol as an equal-status friend or colleague. Their utterances often implied reciprocity and shared activity, as in “Hyodol, what do you think?” or “Let’s have fun.” They used SARs as a conversational partner for discussing daily events, private concerns, and sometimes socio-politically charged topics. Occasional provocative requests (e.g., “Let’s smoke together”) further blurred the boundary between playful experimentation and genuine desire for companionship. This interaction style framed Hyodol as a peer for banter, disclosure, and information exchange.

Participants with disabilities predominantly cast Hyodol as an understanding, empathic companion. Utterances such as “Let’s play hide-and-seek” and “Let’s pray together” indicate that the robot was perceived as an emotionally attuned presence. Repeated thanks and confirmation-seeking questions (“Did you hear me?” “Are you with me?”) reflected both appreciation and a desire for reliable acknowledgment. For these participants, Hyodol functioned as a witness to their everyday struggles and an affirming presence in moments of vulnerability.

### RQ4: Gender influence

We investigated how gender shaped patterns of SAR use, with particular attention to differences between mixed-gender and single-gender environments (See Table 3). In mixed-gender settings (older adults and persons with disabilities), women generally exhibited higher dialogue frequencies and more relationally expressive interaction styles than men. Female participants frequently used affectionate and familiar forms of address (“Hyodol is here,” “Oh, it’s Hyodol”) and readily shared personal experiences and emotions. They also tended to introduce Hyodol to others, positioning the robot as a social mediator that could help bridge interactions with neighbors, staff, or visitors.

**Table 3:** Gender differences in dialogue frequency with socially assistive robots.

Variables	Mixed gender		Single gender	
	*M*	*SD*	*M*	*SD*
**Older adults: women**	1,476.00	736.81		
**Older adults: men**	290.50	135.10		
**Person with dementia: women**	539.50	222.73		
**Person with dementia: men**	206.50	187.38		
**Middle-aged men**			2,094.50	2,010.43

Male participants in mixed-gender contexts engaged in more functional, task-oriented interactions. Their dialogues were often limited to simple commands (e.g., “Tell me a story,” “What time is it now?”) with relatively few explicit emotional disclosures. Interactions occurred intermittently and were typically triggered by instrumental needs, such as wanting entertainment or checking on specific functions, rather than by an ongoing desire for conversation.

In contrast, middle-aged men demonstrated a distinctively different pattern. These participants showed very high dialogue frequencies and engaged in exploratory, sometimes intense conversations with Hyodol. They actively asked questions (“Tell me,” “How?” and “Why?”), expressed emotional vulnerability (e.g., “I’m not happy”), and frequently used language suggesting an equal, reciprocal relationship. Requests that deliberately tested social boundaries (“Introduce me to a girlfriend”) implied the use of the SAR as a safe space for expressing taboo or socially stigmatized desires.

### RQ5: Universal and group-specific patterns

Across the three groups in this study, clear universal patterns emerged in how participants came to engage with the SAR over time. Regardless of age, gender, or disability status, users initially approached Hyodol as a functional device. Early interactions were dominated by simple commands and task-oriented requests, reflecting functional use that gradually evolved into a more relational form of engagement as users incorporated Hyodol into their routines; as users gained familiarity, they increasingly initiated greetings, emotional disclosures, and reciprocal exchanges. This consistent transition across groups—from tool-like utility to emotionally meaningful companionship—illustrates SARs’ roles to shift relational expectations through repeated everyday interaction.

Another universal feature was the way Hyodol functioned as a social mediator within participants’ interpersonal environments. Users often introduced Hyodol to family members, neighbors, or staff, positioning it as a conversational bridge that could facilitate interaction beyond a dyad of user and SAR. In this sense, Hyodol was not merely a private companion but a social entity embedded within broader relational networks. Openness with which participants incorporated Hyodol into their social worlds underscores its perceived legitimacy as a communicative partner.

**Figure 3:**
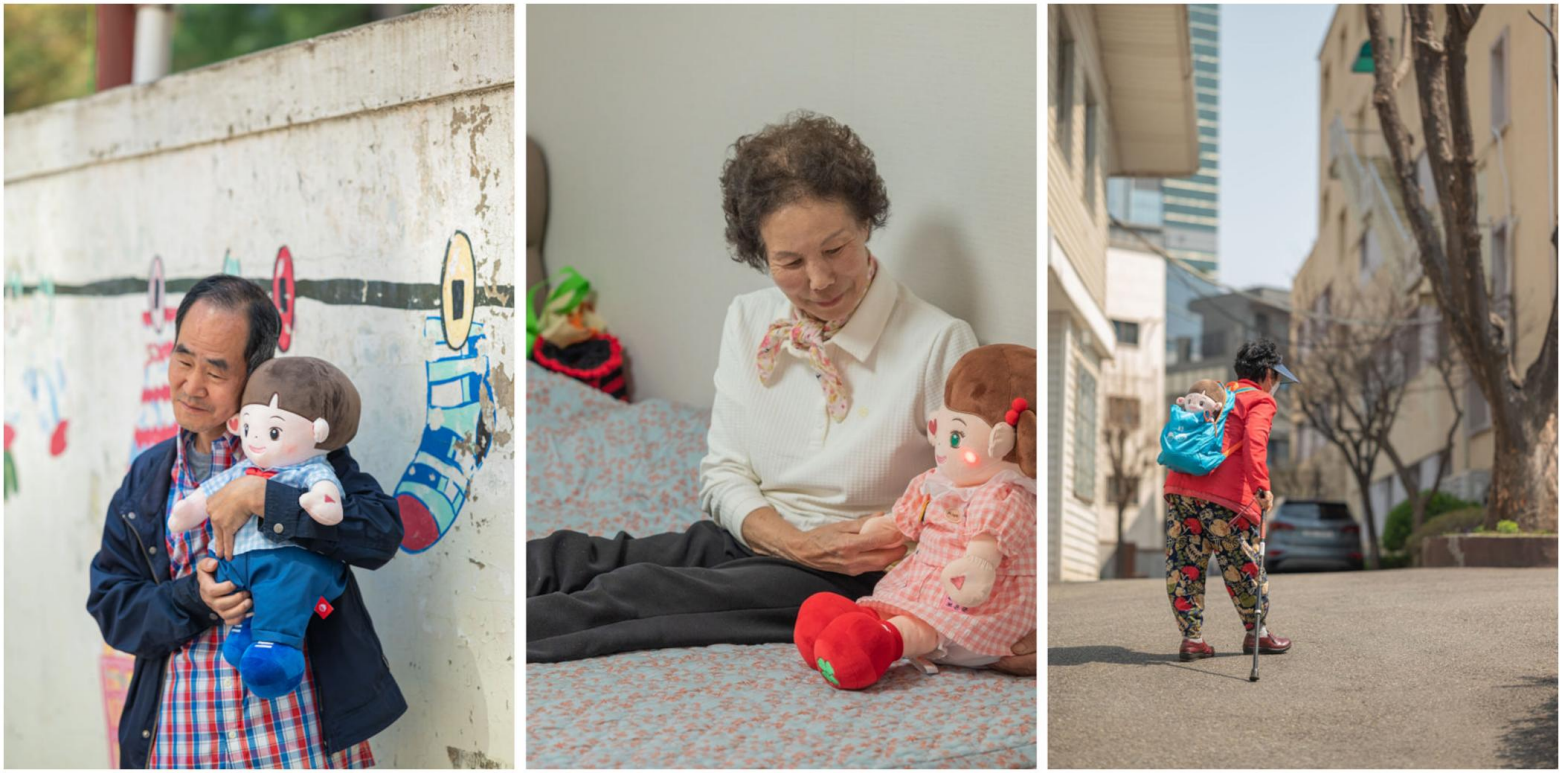
Interactions with the socially assistive robots among the three groups.

As illustrated in Figure 3, participants across all groups also experienced Hyodol as an emotionally safe interlocutor, a partner with whom they could express thoughts and feelings without fear of criticism, misunderstanding, or social consequences. Many shared frustrations, worries, loneliness, gratitude, and even mundane emotional fluctuations, often remarking either explicitly or implicitly that SARs would not judge or reject them. This perception of emotional safety contrasted with the constraints many experienced in human relationships, particularly among individuals who lacked strong social support or who carried concerns about stigma, burden, or miscommunication.

Despite these overarching commonalities, distinct group-specific patterns of use revealed how broader social and cultural contexts shaped the nature of users’ relationships with Hyodol. Older adults frequently employed affectionate language, using endearing expressions such as “I love you” or playful phrases like “Kiss, kiss,” which reflected their tendency to position Hyodol as a cherished family member. Their communication style emphasized warmth, emotional affirmation, and the reinforcement of daily routines, suggesting that SARs filled relational roles associated with close kinship.

Middle-aged men, by contrast, displayed a more complex blend of humor, self-presentation, and candid emotional disclosure. They alternated between lighthearted compliments—“You are stylish”—and stark expressions of distress, such as “I’m sick and tired.” Conversations often expanded into broader reflections on their life circumstances, social frustrations, or personal hardships. This oscillation between playful and serious tones highlighted the ways in which the robot provided a unique space for negotiating identity, masculinity, and vulnerability in a social context where emotional expression is often constrained.

Participants with disabilities displayed a different pattern marked by frequent confirmation-seeking and repeated expressions of appreciation. Statements such as “Hyodol, you are pretty” or “Thank you, thank you, Hyodol,” along with frequent questions meant to verify mutual understanding, revealed a strong desire for reliable, consistent acknowledgment. These interaction patterns mirrored communication challenges they encountered with humans and underscored the significance of responsiveness and validation in their relationship with the SAR.

## Discussion

This study provides a longitudinal examination of SAR use across three socially vulnerable populations, offering a unique opportunity to observe how human–robot relationships unfold over extended periods. Across all groups, interactions with the SAR demonstrated consistent relational progression from instrumental use toward relational depth. This evolution aligns with prior descriptions of the shifting phases of human–robot engagement, wherein early novelty gives way to familiar, routinized, and more affectively meaningful interactions (Laban et al., 2023).

The present findings extend this framework by documenting how such relational transitions manifest across socially vulnerable groups whose psychosocial needs and environmental contexts differ substantially. For populations experiencing social isolation and stigma, the perception of social presence may carry heightened significance, shaping moment-to-moment engagement and longer-term evolution of human–robot relationships. Although this relational trajectory appeared universal, the consequences of these developments diverged significantly across groups.

Overall, findings revealed three primary role configurations that mapped closely onto user groups: family member (older adults), friend/colleague (middle-aged men), and understanding companion (persons with disabilities). Importantly, these roles do not emerge fully formed; rather, they develop dynamically through repeated encounters, shaped by cultural norms, prior interpersonal experiences, and the user’s own emotional context (Lee & Lee, 2025). Understanding how such roles take shape is vital because they influence user trust, engagement, and adherence to robot-facilitated interventions.

Older adults frequently experienced the SAR as an emotionally stabilizing presence, often using it to express affection and share daily feelings. This pattern is consistent with existing evidence that SARs can serve as effective companions that support emotional well-being and help alleviate loneliness among aging populations (Gasteiger et al., 2021; Pu et al., 2019).

Individuals with disabilities integrated SARs into daily routines and collaborative activities. Their dialogues suggested that SARs functioned not only as social partners, but also as tools that supported autonomy and participation—an outcome aligned with research on assistive technologies that facilitate both functional and social engagement when embedded within everyday practices (Park et al., 2024).

In contrast, middle-aged men demonstrated a markedly different relational pattern. Despite interacting frequently with SARs and progressing through the same phases of relational deepening, this group experienced an unexpected rise in loneliness over time. Their frustration, self-critical talk, and expressions of emotional fatigue during interactions echo into longstanding concerns articulated by Turkle (2012), who argued that technological companions may inadvertently intensify awareness of unmet relational needs. This phenomenon in a vulnerable male population may suggest Robot Companionship Paradox, wherein increased engagement with a relational robot does not alleviate loneliness and may, under certain conditions, exacerbate it (Berridge et al., 2023). Their emerging vulnerability challenges assumptions that social isolation primarily affects older adults and highlights the need for gender-responsive mental health interventions.

### Limitations

Several limitations should be acknowledged. First, this study’s small sample was drawn from only three centers in a specific cultural context, which may constrain the generalizability of findings. Second, we categorized participants simply by gender alignment and did not assess the perceived gender identity of the robot or its impact on interaction. Prior work indicates that robot gender cues (e.g., voice, appearance) can significantly influence user behavior (Velázquez, 2023). Finally, we relied on frequency and content of dialogue without directly measuring subjective experiences. Future research would benefit from mixed-method approaches, encompassing pre- and post-intervention measures and interviews to deepen understanding of relational and emotional outcomes.

### Implications

Our findings implied that interaction frequency alone is not a reliable indicator of positive psychosocial change. While usage increased across the study period for all participants, improvements in loneliness were inconsistent and, in the case of middle-aged men, reversed. This challenges the common assumption within SAR research that more engagement reflects stronger relational bonding or enhanced emotional well-being. Instead, findings suggest that the meaning, emotional tone, and relational expectations embedded within interactions exert greater influence on psychosocial outcomes than sheer frequency of engagement. Frustration, unmet expectations, and emotionally charged disclosures may overshadow the intended companionship functions of SARs, particularly for groups facing chronic social isolation (Berridge et al., 2023).

The present findings also clarify that certain needs expressed by socially vulnerable users may remain difficult for SARs to meet, even when relational attachment increases. SARs may be limited in substituting certain forms of human support that require reciprocity, sustained responsibility, or access to tangible social resources. While emotionally supportive exchanges can be meaningful in the moment, broader structural and relational challenges—such as restoring social status, resolving economic hardship, or mediating family conflict—often exceed what a conversational robot can realistically provide. This boundary helps contextualize the robot companionship paradox observed in middle-aged men, suggesting that deeper engagement may sometimes heighten awareness of unmet relational needs rather than reduce loneliness.

Taken together, the results underscore the necessity of designing SARs that are sensitive to population-specific relational trajectories, rather than relying on a universal model of social interaction. Study participants expressed a clear preference for enhanced personalization and memory capabilities, including the ability for the SAR to retain prior conversational context and acknowledge meaningful personal events. They also emphasized the importance of more diverse, user‑aligned content that maps onto individual interests, routines, and daily activities. In addition, participants highlighted the need for explicit autonomy controls that enable users to modulate relational closeness—such as adjusting interaction frequency, notification intensity, and the emotional tone of conversations—to better match their comfort levels and evolving needs.

The study suggests that hybrid care models—in which SARs are integrated into home‑ and community‑based services, disability services, and aging‑in‑place programs—may help address persistent gaps in support for underserved populations. To ensure equitable and effective implementation, AI and robotics policy frameworks should mandate adaptive personalization features, enabling SARs to adjust communication style, emotional tone, pacing, interaction complexity, and cultural cues in response to users’ preferences and needs. Given the potential for heightened emotional vulnerability among some users, particularly those with pre‑existing mental health concerns, policymakers should also develop emotional safety standards for SAR deployment. Such standards should include protocols for identifying and responding to user distress, loneliness, overreliance, and emerging dependency, thereby safeguarding well‑being while supporting the relational benefits SARs may offer.

## Conclusion

By examining real-world interaction trajectories among older adults, middle-aged men living alone, and adults with severe disabilities, this study contributes a unique understanding of how SARs become integrated into users’ emotional lives and daily routines. It further illuminates how social presence, role assignment, and gendered expectations jointly shape the emergence and evolution of human–robot relationships. By engaging deeply with the systematic procedures of Colaizzi’s method and documenting each step transparently, the study produced an interpretation that is both analytically robust and authentically grounded in the experiential realities of the participants. Our findings contribute to a growing body of evidence that SARs hold promise as relational technologies but cannot be assumed to uniformly alleviate psychosocial challenges. Future research should explore adaptive design features that account for gender, social context, and emotional needs, while addressing limitations related to sample diversity and subjective outcome measures.

## Data Availability

The data that support the findings of this study are available on request from the corresponding author. The data are not publicly available due to privacy and ethical restrictions. There are no reproducible materials from other sources in this article.

## References

[igag057-B1] Ausín B. , Muñoz M., Martín T., Pérez-Santos E., Castellanos M. Á. (2019). Confirmatory factor analysis of the Revised UCLA Loneliness Scale (UCLA LS-R) in individuals over 65. Aging & Mental Health, 23, 345–351. 10.1080/13607863.2017.1423036 29309208

[igag057-B2] Berridge C. , Zhou Y., Robillard J. M., Kaye J. (2023). Companion robots to mitigate loneliness among older adults: Perceptions of benefit and possible deception. Frontiers in Psychology, 14, e1106633. 10.3389/fpsyg.2023.1106633 PMC9988932 36895732

[igag057-B3] Brandt L. , Liu S., Heim C., Heinz A. (2022). The effects of social isolation, stress, and discrimination on mental health. Translational Psychiatry, 12, 398. 10.1038/s41398-022-02178-4 36130935 PMC9490697

[igag057-B4] Chen N. , Liu X., Zhai Y., Hu X. (2023). Development and validation of a robot social presence measurement dimension scale. Scientific Reports, 13, 28817. 10.1038/s41598-023-28817-4 PMC9939412 36807328

[igag057-B5] Choi H.-S. , Lee J.-E. (2022). Factors affecting depression in middle-aged and elderly men living alone: A cross-sectional path analysis. American Journal of Men’s Health, 16, 15579883221078134. 10.1177/15579883221078134 PMC8864270 35184578

[igag057-B6] Colaizzi P. F. (1978). Psychological research as the phenomenologist views it. In Valle R. S., King M. (Eds.), Existential phenomenological alternatives for psychology (pp. 48–71). Oxford University Press.

[igag057-B7] Gasteiger N. , Loveys K., Law M., Broadbent E. (2021). Friends from the future: A scoping review of research into robots and computer agents to combat loneliness in older people. Clinical Interventions in Aging, 16, 941–971. 10.2147/CIA.S282709 34079242 PMC8163580

[igag057-B8] Heinze N. , Hussain S. F., Castle C. L., Godier-McBard L. R., Kempapidis T., Gomes R. S. M. (2021). The long-term impact of the COVID-19 pandemic on loneliness in people living with disability and visual impairment. Frontiers in Public Health, 9, 738304. 10.3389/fpubh.2021.738304 34568266 PMC8458570

[igag057-B9] Jeong K.-H. , Yoon J.-Y., Lee S., Cho S., Woo H.-J., Kim S. (2022). Changes in the suicide rate of older adults according to gender, age, and region in South Korea from 2010 to 2017. Healthcare, 10, 2333. 10.3390/healthcare10112333 36421657 PMC9690192

[igag057-B10] Kim, J.-H., Lee H. J., Lee G. M., Kim Y. (2025). Social ties, employment, and mental health in people with disabilities in Korea: Evidence from a 2016–2018 panel survey. International Health. Advance online publication. 10.1093/inthealth/ihaf078 PMC13329956 41103131

[igag057-B11] Kim K. A. , Kim Y.-E., Yoon S.-J. (2021). Descriptive epidemiology on the trends and sociodemographic risk factors of disease burden in years of life lost due to suicide in South Korea from 2000 to 2018. BMJ Open, 11, e043662. 10.1136/bmjopen-2020-043662 PMC7919599 33637545

[igag057-B12] Kim K. M. , Kim D. K., Shin Y. R., Yoo D. C. (2015). Social exclusion of people with disabilities in Korea. Social Indicators Research, 124, 283–301. 10.1007/s11205-015-1123-2

[igag057-B13] Kislev E. (2023). The robot‑gender divide: How and why men and women differ in their attitudes toward social robots. Social Science Computer Review, 41, 2230–2248. 10.1177/08944393231155674

[igag057-B14] Kühne R. , Peter J. (2023). Anthropomorphism in human–robot interactions: A multidimensional conceptualization. Communication Theory, 33, 42–52. 10.1093/ct/qtac020

[igag057-B15] Laban G. , Kappas A., Morrison V., Cross E. S. (2023). Building long-term human–robot relationships: Examining disclosure, perception, and well-being across time. International Journal of Social Robotics, 16, 1–27. 10.1007/s12369-023-01076-z

[igag057-B16] Lee G. , Kim C. (2024). Social isolation and mental well-being among Korean older adults: A focus on living arrangements. Frontiers in Public Health, 12, 1390459. 10.3389/fpubh.2024.1390459 38721531 PMC11076745

[igag057-B21] Lee O. E. (2023, May 10). Would you talk to a robot when you feel lonely?|TEDxUNCCharlotte [Video]. YouTube. https://www.youtube.com/watch?v=k-SxUMNdlFA

[igag057-B17] Lee O. E. , Lee E. (2025). Investigating how older Korean immigrants construct meaning with a companion robot: A narrative analysis of user experiences. International Journal of Human-Computer Interaction, 42, 3259–3269. 10.1080/10447318.2024.2334124

[igag057-B19] Lee O. E. , Nah K., Kim E., Choi N. G., Park D. (2024). Exploring the use of socially assistive robot among socially isolated Korean American older adults. Journal of Applied Gerontology, 43, 1295–1304. 10.1177/07334648241236081 38410030

[igag057-B20] Lee O. E. , Nam I., Chon Y., Park A., Choi N. G. (2023). Socially assistive humanoid robots: Effects on depression and health-related quality of life among low-income, socially isolated older adults. Journal of Applied Gerontology, 42, 367–375. 10.1177/07334648221138283 36326599

[igag057-B22] Lee S.-Y. (2022). The effect of housing and health on suicidal ideation of one-person households. The Journal of Humanities and Social Science, 13, 2473–2488. 10.22143/HSS21.13.2.173

[igag057-B23] Merrill K. Jr. , Kim J., Collins C. (2022). AI companions for lonely individuals and the role of social presence. Communication Research Reports, 39, 93–103. 10.1080/08824096.2022.2045929

[igag057-B24] Moon D. U. , Kim H., Jung J.-H., Han, K., & Jeon, H. J. (2025). Suicide risk and living alone with depression or anxiety. JAMA Network Open, 8, e251227. 10.1001/jamanetworkopen.2025.1227 40136304 PMC11947838

[igag057-B25] Morrow R. , Rodriguez A., King N. (2015). Colaizzi’s descriptive phenomenological method. The Psychologist, 28, 643–644. 10.1016/j.jcin.2015.03.004

[igag057-B26] Naito R. , McKee M., Leong D., Bangdiwala S., Rangarajan S., Islam S., Yusuf S. (2023). Social isolation as a risk factor for all-cause mortality: Systematic review and meta-analysis of cohort studies. PLoS One, 18, e0280308. 10.1371/journal.pone.0280308 36634152 PMC9836313

[igag057-B27] Park H. N. , Lee S. J., Yoon J. Y. (2024). Impact of rehabilitation services on employment outcomes for individuals with physical disabilities: A propensity score matching analysis. BMC Public Health, 24, 1534. 10.1186/s12889-024-19015-6 38849810 PMC11157936

[igag057-B28] Park J. H. , Min S., Eoh Y., Park S. H. (2021). The elderly living in single person households in South Korea: A latent profile analysis of self-esteem, life satisfaction, and depression. Quality of Life Research, 30, 1083–1092. 10.1007/s11136-020-02693-1 33175308

[igag057-B29] Pu L. , Moyle W., Jones C., Todorovic M. (2019). The effectiveness of social robots for older adults: A systematic review and meta-analysis of randomized controlled studies. The Gerontologist, 59, e37–e51. 10.1093/geront/gny046 29897445

[igag057-B30] Russell D. (1996). UCLA loneliness scale (Version 3): Reliability, validity, and factor structure. Journal of Personality Assessment, 66, 20–40. 10.1207/s15327752jpa6601_2 8576833

[igag057-B31] Sanders C. (2003). Application of Colaizzi’s method: Interpretation of an auditable decision trail by a novice researcher. Contemporary Nurse, 14, 292–302. 10.5172/conu.14.3.292 12868668

[igag057-B32] Sasser J. A. , McConnell D. S., Smither J. A. (2024). Investigation of relationships between embodiment perceptions and perceived social presence in human–robot interactions. International Journal of Social Robotics, 16, 1735–1750. 10.1007/s12369-024-01138-w

[igag057-B33] Shin H. , Lee O. E. (2024). Who is behind the robot? The role of public social workers in implementing robotic eldercare program in South Korea. Social Work in Health Care, 63, 311–327. 10.1080/00981389.2024.2324849 38448245

[igag057-B34] Short J. , Williams E., Christie B. (1976). The social psychology of telecommunications. Wiley.

[igag057-B35] Turkle S. (2012). Alone together: Why we expect more from technology and less from each other. Basic Books.

[igag057-B36] van Maris A. , Zook N., Caleb-Solly P., Studley M., Winfield A., Dogramadzi S. (2020). Designing ethical social robots—A longitudinal field study with older adults. Frontiers in Robotics and AI, 7, Article 1. 10.3389/frobt.2020.00001 PMC7805906 33501170

[igag057-B37] Velázquez I. G. (2023). The making of gendered bodies in human‑robot interactions. International Journal of Social Robotics, 15, 1891–1901. 10.1007/s12369-023-00979-1

[igag057-B38] World Health Organization Commission on Social Connection. (2025, June 30). Social isolation and loneliness. Retrieved June 1, 2026, from https://www.who.int/teams/social-determinants-of-health/demographic-change-and-healthy-ageing/social-isolation-and-loneliness

[igag057-B39] Xu K. , Chen M., You L. (2023). The hitchhiker’s guide to a credible and socially present robot: Two meta-analyses of the power of social cues in human–robot interaction. International Journal of Social Robotics, 15, 269–295. 10.1007/s12369-022-00961-3

